# Adherence to the Planetary Health Diet and Its Association with Diet Quality in the Young Adult Population of Türkiye: A Large Cross-Sectional Study

**DOI:** 10.3390/nu16060868

**Published:** 2024-03-17

**Authors:** Hande Mortaş, Semra Navruz-Varlı, Saniye Bilici

**Affiliations:** Department of Nutrition and Dietetics, Faculty of Health Sciences, Gazi University, Ankara 06490, Türkiye; semranavruz@gazi.edu.tr (S.N.-V.); sgbilici@gazi.edu.tr (S.B.)

**Keywords:** Healthy Eating Index, Planetary Health Diet Index, young adults, university students

## Abstract

To advance both human health and environmental sustainability, it’s crucial to assess the adaptation to new dietary trends emerging in this field. This study aimed to explore the relationship between diet quality and the principles of planetary health diet in young adults studying at university. This cross-sectional study consisted of 945 young adults with a mean age of 20.1 ± 1.34 y (582 females, 363 males). A questionnaire form containing socio-demographic information (age, gender, education level), anthropometric measurements (body weight and height), and a 24 h dietary record form for three consecutive days was applied. The scores of the Planetary Health Diet Index (PHDI) and the Healthy Eating Index-2020 (HEI-2020) were calculated according to the dietary records. The mean total scores of the PHDI and HEI-2020 were 59.9 ± 14.16 and 54.2 ± 10.87, respectively. The association between the HEI-2020 score and the PHDI score was significant (*p* = 0.003). A one-unit increase in the unadjusted HEI-2020 score caused a 0.429 unit decrease in the PHDI score (95% CI: −0.709; −0.149). The findings underscore the imperative for targeted interventions and educational programs to enhance the PHDI and HEI-2020 scores, promoting individual well-being and environmental sustainability in the university.

## 1. Introduction

Unhealthy diets rank among the top ten risk factors contributing to the global burden of disease, with dietary factors being implicated in one out of every five deaths worldwide [1]. Diseases relating to unbalanced diets diseases are high and increasing worldwide. Deaths associated with poor diets have increased by 15% from 2010–2022, and deaths from diet-related non-communicable diseases (NCDs)—more than 12 million—are responsible for 26% of all adult deaths [2]. As is clear from these data, the adverse effects of unhealthy diets on human health have reached profound proportions and are increasing daily.

The adverse effects of unhealthy diets on the environment, in addition to the adverse effects on human health, have been discussed intensively in recent years. In the many production and processing steps that foods go through from field to fork, natural resources are used intensively, and the environment is damaged. For example, food production contributes 21–37% of greenhouse gas emissions (GHGEs), occupies more than 40% of the land, and uses 70% of freshwater resources globally [3].

Due to limited resources, environmental damage, and unsustainable production and consumption models, existing food systems must be more comprehensive to provide nutrient-rich food that will encourage adequate and balanced nutrition [4]. As a result of the implementation and dissemination of healthy and sustainable diets, GHGEs from food systems will be reduced, and public health will be improved [5]. So, particular dietary models are required to promote optimal health and sustainability. 

According to the Food and Agriculture Organization (FAO) and the World Health Organization (WHO), healthy diets will be sustainable when they support all dimensions of individuals’ health and well-being [4]. In this context, a sustainable diet model called the “Planetary Health Diet (PHD)”, which aims for good health for both society and the planet, is recommended by the EAT–Lancet Commission [6]. The “Planetary Health Diet Index (PHDI)” was developed with the basic principles of the EAT-Lancet diet in mind, and higher PHDI scores were associated with an increase in overall diet quality, a decrease in GHGEs, and a reduction in obesity and obesity indicators in the Brazilian population [7,8]. In a large sample study, the average PHDI total score was only 30.6% of the maximum possible score [9]. 

University students, being representative of the young age demographic, play a pivotal role in shaping societal dietary habits, which, from a life cycle standpoint, can significantly impact nutritional status and health later in life. Determining the diet quality of young females and males is crucial due to their future role as food preparers for household members in their private lives [10,11]. In addition, the university students who constitute the sample group in this study have a high potential to contribute positively to the diet quality of the target group they serve during their professional practice after graduating from university. The college entry phase is when young adults experience a greater sense of freedom and independence and take on the responsibility of selecting, purchasing, preparing, and cooking food, often alone [12,13,14]. The eating habits of university students generally depend on the course schedules that students have to comply with (face-to-face/online classes) and the availability of food on or near the university campus or dormitory [13]. As a result of the spread of fast-food-style foods, because they are fast and cheap, and the lack of suitable dining areas, students often skip meals, reduce food variety, and consume unhealthy snacks more frequently, which causes obesity [12,15,16]. It is essential to understand the current situation of young people during their university years regarding issues such as healthy nutrition, diet quality, and nutritional practices for planetary health. This will then serve as a guide to taking proactive measures to prevent nutrition-related health problems that may occur in middle and old age in the future and to protect the planet’s health. So, given the prevalence of the obesity epidemic among university students, this study aimed to determine compliance with the PHDI and its relationship with diet quality among young adults in Türkiye. 

## 2. Materials and Methods

### 2.1. Participants and Study Design

This cross-sectional study consisted of 945 young adults (582 females, 383 males) studying at universities in Ankara between February 2022 and June 2022, which included the winter, spring, and summer seasons. So, a general perspective has been provided in terms of seasonality. In order to determine the sample size, the analysis was performed by taking alpha (α) = 0.05 and power (1-β) = 0.95 via the G*Power 3 software program. The number of samples determined as a result of the analysis was 920. The inclusion criteria of the participants in the study were in the age range of 19–30, being a volunteer and university student, not following a special diet or eating model, not having any chronic disease such as cardiovascular diseases, diabetes, food allergies that require a special diet, and not using a nutritional supplement, protein powder, etc. Individuals with a daily energy intake of <600 or >3500 kcal according to their 24 h dietary records and who were pregnant or lactating were excluded from the study. 

Ethical approval was obtained from the Ethical Committee of Gazi University (Date: 11 January 2022 No: 2022—052). In addition, written informed consent was obtained from the participants in the study. The research was carried out following the Declaration of Helsinki.

### 2.2. Data Collection Tools

In the study, questionnaire forms containing socio-demographic information (age, gender, education level) and 24 h dietary record forms for three consecutive days were completed by the researchers through face-to-face interviews. The researchers measured body weight and height using a Tanita in Tokyo, Japan BC 532 Innerscan scale and stadiometer, respectively. Body mass index (BMI) was defined as body weight in kg divided by the square of the height in m (kg/m^2^) [17].

A nutritional assessment was made using the 24 h dietary record data for three consecutive days from all the participants by the researchers. The participants were asked to record everything consumed (including foods, beverages, sauces, and condiments) for three consecutive days after the interview. The contents of the dishes consumed by the individuals were calculated using the book *Standard Food Recipes* [18]. The “Food and Nutrition Photo Catalog” was used so that individuals could write down the portion sizes of the meals they consumed. Through this catalog, participants reported the portion sizes of the meals they consumed according to the meal samples in the photographs [19]. The data were analyzed using the BeBiS program for total energy and nutrient intake (BeBiS, 7.2 version). 

### 2.3. Instruments to Assess Diet Quality 

The Planetary Health Diet Index (PHDI) was developed by Cacau et al. (2021) from the EAT–Lancet Commission’s dietary recommendations [7]. The components of this index are scored between 10 and 5 points, with a total score between 0 and 150. The 16 diet components were evaluated based on food records. The Participants were divided into tertiles based on their total PHDI score.

The index components and the maximum possible points in parentheses are red meat (10 points), nuts and peanuts (10 points), legumes (10 points), chicken and its substitutes (10 points), fish and seafood (10 points), eggs (10 points), fruit (10 points), vegetables (10 points), the ratio of dark green leafy vegetables to other vegetables (5 points), the ratio of red vegetables to other vegetables (5 points), whole grains (10 points), milk and its products (10 points), unsaturated fats (10 points), animal fats (10 points), and added sugars (10 points).

The Healthy Eating Index was created by the United States Department of Agriculture (USDA) in 1995, based on the American Dietary Guidelines [20,21]. According to the American Dietary Guidelines, it was updated in 2005, 2010, and 2015. The components and standards did not change between the HEI-2015 and HEI-2020. The HEI-2020 maintains complete alignment with the HEI-2015 in its 13 components and scoring standards, despite the index being renamed to emphasize its correlation with the latest 2020–2025 Dietary Guidelines for Americans.

HEI-2020, which was used in this study, consists of 13 components, 9 of which should be consumed, and 4 of which should be consumed less. Nine of these components that are desired to be consumed are total fruit, whole fruits, total vegetables, green leafy and fresh legumes, whole grains, dairy products, protein foods, seafood and vegetable proteins, and fatty acids. The four that should be consumed in moderation are processed grains, sodium, added sugar, and saturated fats. In the index, the scores of the components range from 0 to 5 or 0 to 10, with low scores reflecting malnutrition and high scores reflecting good nutrition [22]. 

### 2.4. Statistical Analysis

Continuous variables were expressed as arithmetic means with standard deviations and categorical variables as percentages. The age, BMI, HEI-2020 total score, PHDI total score, tertile percentages of indices, and dietary energy, macronutrients, and micronutrients were compared according to gender. The participants’ total and subgroup indices scores were categorized according to BMI groups. Chi-square analysis facilitated the comparison of qualitative data to identify group differences. The *t*-test, Mann–Whitney U test, one-way ANOVA, or Kruskal–Wallis test were employed for comparisons in independent groups. The post hoc analysis involved the application of Bonferroni correction for handling multiple pairwise comparisons.

Furthermore, linear regression was used to determine factors related to the PHDI score to explain the relationships between observable associations. The HEI-2020 total score and subgroup scores were selected as predictors, and models 1 and 2 were adjusted for age, gender (0 for females and 1 for males), and total energy intake (kcal/d) for age and gender (0 for females and 1 for males), respectively. The results were interpreted with 95% confidence. The IBM SPSS Statistics 28.0.1.0 program was used for statistical analysis, and significance was evaluated at *p* < 0.05 level.

## 3. Results

The general characteristics, HEI-2020 and PHDI scores, and dietary intakes according to gender are shown in Table 1. The participants’ mean age and BMI were 20.1 ± 1.34 y and 22.0 ± 3.05 kg/m^2^, respectively. The mean BMI of the females (22.4 ± 3.36) was higher than that of the males (21.5 ± 2.40; *p* < 0.001). According to the BMI categories, underweight (12.4% and 2.9%, respectively) and overweight and obese (18.6% and 9.9%, respectively) showed higher percentages in females than in males (*p* < 0.001). The females’ mean HEI-2020 score was higher than the males’ (55.1 ± 10.76 and 52.8 ± 10.90, respectively, *p* = 0.002). According to the tertiles of HEI-2020, the percentages in the average (T_2_: 33.0% and 32.8%, respectively)) and high (T_3_: 36.9% and 29.5%, respectively) groups were higher in the females than the males (*p* = 0.022). The males were in the low diet quality group (T_1_) at a higher percentage (37.7% and 30.1%, respectively, in males and females). There was no statistically significant difference between genders in the PHDI mean score and the percentage distributions in the PHDI categories. There was no significant difference in the dietary energy intake of individuals between genders, and the mean dietary energy intake of the participants was 1903.6 ± 87.43 kcal. The percentages of the mean contribution of individuals’ dietary carbohydrate, protein, and fat intakes to daily energy intake were 42.3 ± 9.75, 14.9 ± 4.25, and 42.7 ± 8.84, respectively. The percentage of protein intake from macronutrients contributing to daily energy was higher in the females than in the males (15.1 ± 4.56 and 14.5 ± 3.65, respectively; *p* = 0.013). The mean cholesterol intake of the males was 328.5 ± 21.14 mg, while the mean cholesterol intake of the females was 298.9 ± 24.54 mg (*p* = 0.048). The daily fiber intake of the participants was 22.5 ± 9.93 g, and there was no difference between genders. The individuals’ daily micronutrient intakes in their diets are also shown in Table 1. There was no significant difference between genders in these values. 

The mean and standard deviation values of the scores received by individuals from HEI-2020 and PHDI and the subcomponents of these indexes according to the BMI category are presented in Table 2. There were no significant differences among BMI categories in the HEI-2020 score. It was revealed that the scores of whole grains, dairy, the MUFA/PUFA ratio, and saturated fatty acids, which are HEI-2020 subcategories, differed between BMI categories. The whole grains score was lower in normal weight (1.1 ± 2.83) than in underweight (1.9 ± 3.73) and overweight/obese individuals (1.8 ± 3.46; *p* = 0.006). The dairy score was higher in the normal weight group (4.5 ± 2.92) than in the overweight and obese (3.6 ± 2.72; *p* = 0.001). The mean score of the MUFA/PUFA ratio was higher in the underweight (0.9 ± 2.89) than in the normal weight (0.3 ± 1.79) and overweight/obese groups (0.2 ± 1.21; *p* = 0.005). The mean score of saturated fatty acids was higher in the underweight group (4.7 ± 3.7) than in the normal weight group (3.5 ± 1.79; *p* = 0.007). There were no significant differences among BMI categories in the PHDI score. It was revealed that the scores of red meat, whole grains, and added sugar, which are HEI-2020 subcategories, differed between BMI categories. The mean score of red meat was higher in the underweight group (4.4 ± 4.95) than in the normal weight group (3.3 ± 4.66; *p* = 0.039). The whole cereals score was lower in the normal weight (0.7 ± 1.81) than in the underweight (1.4 ± 2.95) and overweight/obese individuals (1.2 ± 2.66; *p* < 0.001). The added sugar score was found to be higher in the overweight/obese (6.5 ± 4.09) group than in the underweight (4.4 ± 4.08) and normal weight groups (5.5 ± 4.39; *p* = 0.001).

Linear regression models showing the associations of the HEI-2020 and subcomponent scores with the PHDI scores are shown in Table 3. There was no statistical association between the HEI-2020 and PHDI scores in the models where adjustments were made for participants’ age and gender (Model 2) and total energy intake in addition to age and gender (Model 1; *p* > 0.05). The association between the HEI-2020 score, which was not adjusted for any parameters, and the PHDI score was significant (*p* = 0.003). A one-unit increase in the unadjusted HEI-2020 score caused a 0.429 unit decrease in the PHDI score (*p* = 0.003). The association of the HEI-2020 subcomponent scores, dairy, total vegetables, total fruit, and the MUFA/PUFA ratio, where adjustment was made for gender, age, and total daily energy intake, with the PHDI score was found to be statistically significant. One-unit increases in dairy, total vegetable, and total fruit scores were associated with an increase of 0.130, 0.420, and 0.253 units in the PHDI score, respectively, (*p* values = 0.029; <0.001; and 0.017, respectively). A one-unit increase in the MUFA/PUFA ratio score was associated with a 0.188-unit decrease in the PHDI score (*p* = 0.007).

**Figure 1:**
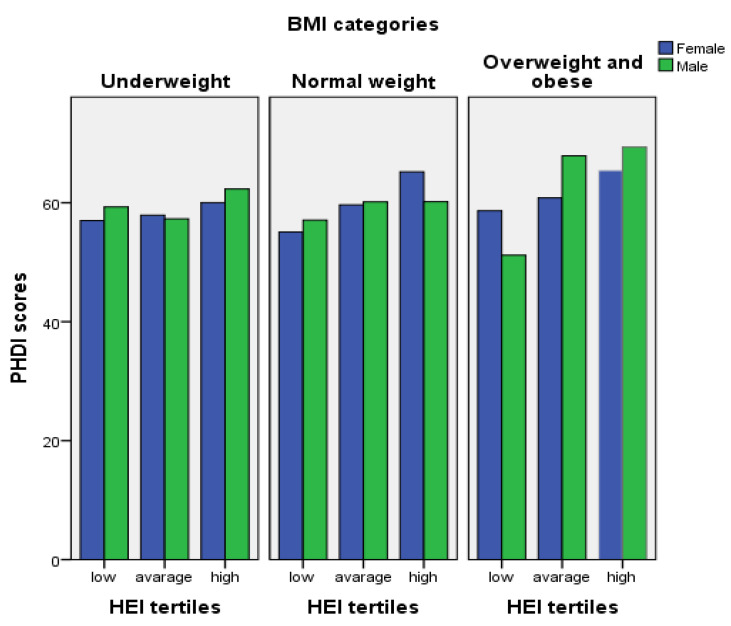
A graph visually summarizing participants’ PHDI scores according to BMI, gender, and HEI tertiles (created from SPSS). BMI: body mass index; HEI: Healthy Eating Index-2020; PHDI: Planetary Health Diet Index.

The graph showing the PHDI score means of the participants according to their gender, BMI category, and HEI tertile is shown in Figure 1. The green bars in the figure represent males. Blue bars represent females. Each beige-filled box displays (1) underweight, (2) normal weight, and (3) overweight and obese groups, respectively. In each beige box, males and females are shown in three categories: low, average, and high; under the tertiles, they are divided according to their HEI-2020 scores. The lengths of the bars vary according to the mean PHDI scores; that is, the vertical axis shows the mean PHDI scores. It was shown that participants in the higher HEI tertile in each BMI category also had higher PHDI score means in both genders. Those in the higher HEI tertile in the overweight/obese category had a higher PHDI mean score than all other categories. The PHDI scores of participants in the average HEI tertile category increased gradually from the underweight to the overweight/obese group. Except for females in the average HEI tertile of the underweight category, in the high HEI tertile of the normal weight category, and the low HEI tertile of the overweight/obese category, the PHDI mean scores were higher in males than in females in all other categories.

## 4. Discussion

In this study, which is the first research conducted on Turkish youth to evaluate the relationship between diet compliance with HEI-2020 and PHDI, it was found that young adults had low HEI-2020 and PHDI scores. While the diet quality of young males was found to be lower than that of females, no significant differences were found in PHDI scores between genders and BMI categories in both scores. There was no relationship between the HEI-2020 total score and the PHDI total score. However, a positive relationship was shown between HEI-2020 subcomponents, including dairy, total vegetables, and total fruit scores, and the PHDI scores. A negative relationship was found between the MUFA/PUFA ratio score, one of the HEI-2020 subcomponents, and the PHDI score.

In this study, females’ HEI-2020 scores were found to be significantly higher than those of males. Similar to this result, dietary patterns seem to be strongly predicted by gender [23,24]. It has been discovered that the eating habits of males and females differ, with males consuming more meat and females consuming more fruit and vegetables. Moreover, Seffen et al. (2023) demonstrated that females were more likely than males to intend to diminish meat intake [25], and another study [26] showed that gender was the most important predictor of reduced meat consumption. Conversely, females tend to be less attached to meat and to have more positive attitudes about plant meals [27]. In support of this information infrastructure, in this study, it is thought that this difference between HEI-2020 scores occurs because females show more vegetable- and fruit-based diet patterns than males, and males, on the contrary, show more animal-based diet patterns. Dietary cholesterol intake of males has also been shown to be higher than that of females, which is another indicator of this finding that reveals differences in nutritional patterns between genders.

In addition to the fact that obesity is known to have adverse effects on environmental sustainability and public health, the unhealthy diet that plays a role in obesity does not coincide with the PHDI model [7,28]. In support of this information, although some studies [8,29,30] associate increased consumption of PHDI-compatible diets with lower BMI, on the contrary, interestingly, there was also a study [9] showing that compliance with sustainable diet models was higher in individuals who were overweight or in the obese category. However, in this study, no statistically significant difference was found in individuals’ HEI-2020 and PHDI scores according to BMI classification. However, similar to other studies [8,29,30], this study showed that the score obtained from the PHDI subcategory evaluating red meat consumption, which is the focus of sustainable diet models, was significantly higher in the underweight category. Interestingly, it has been found that the score of obese and overweight participants from the added sugar subcategory was higher than those in the underweight and normal BMI categories. The single aspect of food intake that our results capture may help partially explain the results’ disparities. Additionally, it is thought that differences between studies may have arisen because a large percentage of participants in this study (74.3%) had normal BMI. 

Although there was no statistically significant relationship between the HEI-2020 score and the PHDI score, the PHDI score was found to be significantly positively correlated with the dairy products, total vegetable, and total fruit intake scores, which are the HEI-2020 subcomponents, as expected. A significant negative relationship was found between the MUFA/PUFA ratio score and PHDI score, one of the HEI-2020 subcomponents. The increased consumption of seafood can explain this relationship. Looking at the calculation details of the scores [9,22], there is a U-shaped relationship between the increase in seafood consumption and the increase in the PHDI score. Therefore, the direction of the relationship would change if the participants had higher amounts of fish consumption. 

When individuals were divided according to BMI categories, it was shown that females in higher tertile categories of HEI-2020 score in all three BMI categories also had higher mean PHDI scores. In males, this situation was shown only in the normal, overweight, and obese categories. Although these increases were shown visually, they were not found to be statistically significant. Finding a positive relationship between the balanced intakes of the components already included in the HEI-2020 score calculation and the PHDI score is inevitable. When examining optimal healthy diets, foods, and dietary patterns, these reference healthy eating patterns emphasizing vegetables, fruits, whole grains, legumes, nuts, and unsaturated fats, and including moderate amounts of seafood and poultry. It advocates for limited or minimal intake of red meat, processed meat, added sugar, refined grains, and starchy vegetables [6]. Therefore, while human health and environmental sustainability are parts of an inseparable whole, they also include subcomponents that overlap and complement each other. It has been reported that win-win diets (healthy and environmentally sustainable) can only be created when a common framework is created by drawing the boundaries of the targets set for these two areas. The index that helps determine the goals of this win-win diet, which has a high adaptability to all food cultures, appears as PHDI [8,9]. In this study, the finding that participants with high HEI-2020 scores in BMI categories had a higher average PHDI score and the finding that scores from HEI-2020 subcomponents were related to the PHDI score supported and encouraged the stated purpose. Similarly, another study has demonstrated that higher PHDI scores were associated with dietary quality [8]. In a new study investigating the compliance of adults with PHDI in Türkiye [28], a positive relationship was found between sustainable and healthy eating behaviors, similar to this study evaluating young adults whose unhealthy eating behaviors are relatively higher. Additionally, according to the other study conducted in Türkiye [28], the PHDI total score (41.6 ± 0.59 and 41.0 ± 0.80 in females and males, accordingly) was detected as lower than this study (60.4 ± 14.48 and 59.2 ± 13.64, in females and males, accordingly). It is thought that the reason for this difference is that the presented study was conducted with university students. However, the participants of the other study [28] included those with less than eight years of education. This inference supports the results of other studies [29,31] showing that increasing the level of education leads to an increase in awareness of the ecological footprint and the effects of consumed diets on the environment.

While we made concerted efforts to gather more precise data from a demographically homogeneous group with balanced age and education level representation, a limitation of the study is that the obtained data might not be broadly applicable to the entire population. Secondly, this cross-sectional study cannot establish a cause-and-effect relationship while assessing the relationship between measured variables. Additionally, nutritional habits exhibit variations not only between countries but also regionally. Therefore, it is crucial to replicate this research in diverse regions and cities nationwide for comprehensive insights. In this study, participants were not questioned about the consumption of bioengineered foods because they are legally prohibited in Türkiye. However, it is recommended that these foods be evaluated in other studies to be planned regarding diet quality. In addition, in order to reduce the bias in individuals’ dietary records, repeating 24 h dietary records for three consecutive days at different times is recommended for future studies. Finally, no blood parameters were evaluated to assess the health status of young adults in this study. Adding this evaluation to the method in future studies will enable the association of participants’ health status with their diet quality. This study boasts several strengths, including its significance in assessing the correlation between planetary health and healthy eating, its substantial sample size, and the distinction of being the first to evaluate the adherence of Turkish youth to the PHDI. Moreover, the index calculation involved collecting dietary intake data through a 24 h record for three consecutive days, a method deemed more accurate than a food frequency questionnaire or a 24 h dietary recall.

## 5. Conclusions

The mean PHDI total score of participants in the high HEI score group was shown to be higher in each BMI category and both genders. Although the education levels of the participants were high, their low compliance with a healthy diet and the EAT-Lancet diet recommendations is a striking result. It reveals the need to revise not only the level but also the content of the education in line with the times. It has become clear that there is a need to add courses to university education curricula that outline how sustainability and healthy nutrition are related and complementary concepts, not only in the fields of health and nutrition but in every field. Raising this awareness in young adults, a group that is just starting to participate in life and meet their shelter, nutrition, and economic needs, is of critical importance in creating healthy generations and using planetary resources efficiently.

## Figures and Tables

**Table 1:** General characteristics, the scores of the Healthy Eating Index-2020, the Planetary Health Diet Index, and dietary intakes according to gender.

General Characteristics	All Participants (*n* = 945)	Females (*n* = 582)	Males (*n* = 363)
Age (y) [$\bar{x}$ ± SD]	20.1 ± 1.34	20.5 ± 1.32	19.4 ± 1.05
		*t* = 13.854	*p* < 0.001
Weight (kg) [$\bar{x}$ ± SD]	58.8 ± 10.03	58.9 ± 10.77	57.8 ± 8.74
		*t* = 0.037	*p* = 0.969
BMI (kg/m^2^) [$\bar{x}$ ± SD]	22.0 ± 3.05	22.4 ± 3.36	21.5 ± 2.40
		*t* = 4.115	*p* < 0.001
BMI categories [*n* (%)]			
Underweight	99 (10.5)	72 (12.4) ^a^	27 (2.9) ^b^
Normal weight	702 (74.3)	402 (69.1) ^a^	300 (82.6) ^b^
Overweight and obese	144 (15.2)	108 (18.6) ^a^	36 (9.9) ^b^
		*χ*^2^ = 21.687	*p* < 0.001
HEI-2020 score [$\bar{x}$ ± SD]	54.2 ± 10.87	55.1 ± 10.76	52.8 ± 10.90
		*t* = 3.172	*p* = 0.002
Tertiles of HEI-2020 [*n* (%)]			
T_1_	312 (33.0)	175 (30.1) ^a^	137 (37.7) ^b^
T_2_	311 (32.9)	192 (33.0) ^a^	119 (32.8) ^b^
T_3_	322 (34.1)	215 (36.9) ^a^	107 (29.5) ^b^
		*χ*^2^ = 7.645	*p* = 0.022
PHDI score [$\bar{x}$ ± SD]	59.9 ± 14.16	60.4 ± 14.48	59.2 ± 13.64
		*t* = 1.206	*p* = 0.228
Tertiles of PHDI [*n* (%)]			
T_1_	312 (33.0)	184 (31.7)	128 (35.1)
T_2_	311 (32.9)	188 (32.2)	123 (30.9)
T_3_	322 (34.1)	210 (36.0)	112 (30.9)
		*χ*^2^ = 2.664	*p* = 0.264
Dietary intake [$\bar{x}$ ± SD]			
Energy (kcal)	1903.6 ± 87.43	1873.9 ± 94.68	1951.2 ± 74.6
		*t* = −1.394	*p* = 0.164
Carbohydrates (% of energy)	42.3 ± 9.75	41.9 ± 9.76	43.2 ± 9.70
		*t* = −1.947	*p* = 0.052
Proteins (% of energy)	14.9 ± 4.25	15.1 ± 4.56	14.5 ± 3.65
		*t* = 2.483	*p* = 0.013
Fats (% of energy)	42.7 ± 8.84	42.9 ± 8.73	42.3 ± 9.02
		*t* = 0.989	*p* = 0.323
Cholesterol (mg)	310.3 ± 23.36	298.9 ± 24.54	328.5 ± 21.14
		*t* = −1.976	*p* = 0.048
Saturated fat (g)	29.1 ± 22.44	28.9 ± 16.61	29.4 ± 13.30
		*t* = −0.412	*p* = 0.680
Fiber (g)	22.5 ± 9.93	22.4 ± 9.83	22.7 ± 10.09
		*t* = −0.588	*p* = 0.556
Riboflavin (mg)	1.4 ± 0.61	1.4 ± 0.66	1.4 ± 0.54
		*t* = −0.743	*p* = 0.458
Niacin (mg)	13.8 ± 10.53	14.1 ± 10.87	13.2 ± 9.96
		*t* = 1.323	*p* = 0.186
Vitamin A (mcg)	468.3 ± 111.4	479.2 ± 139.76	450.7 ± 321.86
		*t* = 0.472	*p* = 0.637
Vitamin B_12_ (mcg)	4.3 ± 6.47	4.3 ± 7.25	4.2 ± 4.99
		*t* = 0.266	*p* = 0.791
Vitamin C (mg)	139.3 ± 10.28	141.3 ± 11.40	135.9 ± 10.42
		*t* = 0.767	*p* = 0.443
Thiamine (mg)	0.9 ± 0.38	0.9 ± 0.40	0.9 ± 0.35
		*t* = 0.026	*p* = 0.979
Folate (mcg)	322.1 ± 140.56	319.9 ± 145.79	325.5 ± 131.86
		*t* = −0.605	*p* = 0.545
Iron (mg)	12.1 ± 5.58	11.9 ± 5.92	12.2 ± 4.98
		*t* = −0.670	*p* = 0.503
Phosphorus (mg)	1172.5 ± 449.27	1165.1 ± 468.49	1184.3 ± 419.97
		*t* = −0.657	*p* = 0.511
Calcium (mg)	705.1 ± 316.07	696.6 ± 319.79	718.7 ± 309.98
		*t* = −1.051	*p* = 0.293
Potassium (mg)	2689.1 ± 1037.18	2709.9 ± 1055.17	2655.7 ± 1008.20
		*t* = 0.789	*p* = 0.430
Zinc (mg)	9.7 ± 5.73	9.7 ± 6.33	9.7 ± 4.61
		*t* = −0.003	*p* = 0.998
Magnesium (mg)	289.4 ± 117.77	288.3 ± 118.50	291.2 ± 116.73
		*t* = −0.372	*p* = 0.710
Copper (mg)	1.7 ± 0.72	1.6 ± 0.74	1.7 ± 0.67
		*t* = −1.014	*p* = 0.311

All percentages are calculated in columns. ^a,b^ represent the statistically significant differences among the line groups at *p* < 0.05. BMI: body mass index; HEI: Healthy Eating Index; PHDI: Planetary Health Diet Index; SD: standard deviation. T_1_: low; T_2_: average; T_3_: high.

**Table 2:** The mean ($\bar{x}$) and standard deviation (±SD) of indices scores according to BMI.

Indices and Their Components	Underweight	Normal Weight	Overweight and Obese	F	*p*
HEI-2020 score [$\bar{x}$ ± SD]	55.1 ± 10.44	54.0 ± 10.93	54.3 ± 10.89	0.428	0.652
HEI-2020 components scores [$\bar{x}$ ± SD]					
Whole grains	1.9 ± 3.73 ^a^	1.1 ± 2.83 ^b^	1.8 ± 3.46 ^a^	5.150	0.006
Refined grains	4.5 ± 4.0	5.1 ± 3.89	5.4 ± 3.88	1.414	0.244
Seafood and plant proteins	3.7 ± 1.75	4.0 ± 1.58	3.9 ± 1.69	2.062	0.128
Sodium	9.3 ± 1.92	8.8 ± 2.59	8.7 ± 2.71	1.665	0.190
Dairy	3.8 ± 3.01 ^a,b^	4.5 ± 2.92 ^a^	3.6 ± 2.72 ^b^	6.635	0.001
Greens and beans	3.6 ± 1.72	3.6 ± 1.84	3.7 ± 1.88	0.390	0.677
Total vegetable	3.1 ± 1.57	3.1 ± 1.59	3.1 ± 1.61	0.014	0.986
Whole fruit	3.9 ± 1.73	3.6 ± 1.99	3.5 ± 1.92	1.403	0.246
Total fruit	2.9 ± 1.68	2.9 ± 1.89	2.8 ± 1.90	0.282	0.754
Added sugar	9.4 ± 1.38	9.6 ± 1.24	9.8 ± 1.15	1.870	0.155
MUFA/PUFA ratio	0.9 ± 2.89 ^a^	0.3 ± 1.79 ^b^	0.2 ± 1.21 ^b^	5.302	0.005
Saturated fatty acids	4.7 ± 3.7 ^a^	3.5 ± 1.79 ^b^	3.8 ± 3.32 ^a,b^	5.030	0.007
PHDI score [$\bar{x}$ ± SD]	58.9 ± 13.25	59.8 ± 13.83	61.6 ± 16.22	1.314	0.269
PHDI components scores [$\bar{x}$ ± SD]					
Red meat	4.4 ± 4.95 ^a^	3.3 ± 4.66 ^b^	3.9 ± 4.86 ^a,b^	3.261	0.039
Nuts and peanuts	5.1 ± 4.69	5.4 ± 4.61	4.5 ± 4.96	2.261	0.105
Legumes	3.0 ± 3.94	3.7 ± 4.21	4.1 ± 4.48	2.032	0.132
Chicken and substitutes	8.3 ± 3.72	7.9 ± 4.02	7.9 ± 3.95	0.395	0.674
Fish and seafood	0.0 ± 0.0	0.0 ± 0.14	0.0 ± 0.0	0.198	0.820
Eggs	0.1 ± 0.98	0.3 ± 1.33	0.2 ± 0.98	1.038	0.355
Fruits	9.2 ± 2.27	9.0 ± 2.49	8.8 ± 2.76	0.663	0.515
Vegetables	8.3 ± 2.89	8.3 ± 2.88	8.3 ± 2.92	0.008	0.992
Whole cereals	1.4 ± 2.95 ^a^	0.7 ± 1.81 ^b^	1.2 ± 2.66 ^a^	8.271	<0.001
Tubers and potatoes	1.1 ± 2.38	1.4 ± 2.62	1.2 ± 2.72	0.538	0.584
Dairy	1.6 ± 2.49	1.6 ± 2.79	1.9 ± 2.86	0.859	0.424
Vegetable oils	3.9 ± 3.45	4.3 ± 3.37	4.1 ± 3.61	0.666	0.514
Animal fats	6.8 ± 4.69	7.1 ± 4.51	7.3 ± 4.44	0.413	0.662
Added sugar	4.4 ± 4.08 ^c^	5.5 ± 4.39 ^b^	6.5 ± 4.09 ^a^	7.053	0.001

^a,b^ represent the statistically significant differences among the line groups at *p* < 0.05. HEI: Healthy Eating Index; MUFA: monounsaturated fatty acids; SD: Standard deviation; PHDI: Planetary Health Diet Index; PUFA: polyunsaturated fatty acids.

**Table 3:** Associations between the Planetary Health Diet Index and the Healthy Eating Index-2020 scores.

	PHDI Scores					
	B	β	*t*	95% CI		*p*
(Constant) ^Model 1^	57.200		75.918	55.722	58.679	<0.001
(Constant) ^Model 2^	59.323		200.525	58.742	59.903	<0.001
(Constant) ^Model 3^	39.999		10.467	32.499	47.498	<0.001
HEI-2020 score ^Model 1^	−0.011	−0.045	−0.198	−0.119	0.098	0.843
HEI-2020 score ^Model 2^	−0.016	−0.183	−1.475	−0.038	0.005	0.141
HEI-2020 score ^Model 3^	−0.429	−0.329	−3.004	−0.709	−0.149	0.003
HEI-2020 components scores ^Model 1^						
Whole grains	0.084	0.098	1.360	−0.037	0.205	0.174
Refined grains	−0.040	−0.059	−0.605	−0.168	0.089	0.545
Seafood and plant proteins	0.064	0.039	0.788	−0.095	0.222	0.431
Sodium	−0.001	−0.001	−0.012	−0.127	0.125	0.991
Dairy	0.130	0.145	2.189	0.013	0.246	0.029
Greens and beans	0.081	0.056	1.080	−0.066	0.227	0.280
Total vegetable	0.420	0.255	5.486	0.270	0.571	<0.001
Whole fruit	−0.075	−0.056	−0.697	−0.287	0.137	0.486
Total fruit	0.253	0.181	2.392	0.046	0.461	0.017
Added sugar	0.067	0.032	0.746	−0.110	0.244	0.456
MUFA/PUFA ratio	−0.188	−0.134	−2.727	−0.323	−0.053	0.007
Saturated fatty acids	−0.005	−0.007	−0.086	−0.121	0.111	0.931

Model 1: Adjusted for sex, age (years), and total energy intake (kcal/d). Model 2: Adjusted for sex and age (years). Model 3: Linear regression model without adjustment. HEI: Healthy Eating Index; MUFA: monounsaturated fatty acids; PHDI: Planetary Health Diet Index; PUFA: polyunsaturated fatty acids.

## Data Availability

The data presented in this study are available on request from the corresponding author. The data are not publicly available due to fact that it is intended to be shared only with researchers working in this field.
